# Altered Functional Connectivity in Children With Low-Function Autism Spectrum Disorders

**DOI:** 10.3389/fnins.2019.00806

**Published:** 2019-08-02

**Authors:** Shoujun Xu, Meng Li, Chunlan Yang, Xiangling Fang, Miaoting Ye, Lei Wei, Jian Liu, Baojuan Li, Yungen Gan, Binrang Yang, Wenxian Huang, Peng Li, Xianlei Meng, Yunfan Wu, Guihua Jiang

**Affiliations:** ^1^The Second School of Clinical Medicine, Southern Medical University, Guangzhou, China; ^2^Department of Medical Imaging, Guangdong Second Provincial General Hospital, Guangzhou, China; ^3^Department of Radiology, Shenzhen Children’s Hospital, Shenzhen, China; ^4^Department of Hematology and Oncology, Shenzhen Children’s Hospital, Shenzhen, China; ^5^Department of Children Healthcare, Shenzhen Children’s Hospital, Shenzhen, China; ^6^Network Center, Air Force Medical University, Xi’an, China

**Keywords:** autism spectrum disorders, spatial patterns, functional connectivity, independent component analysis, Pearson’s correlation analysis

## Abstract

Neuroimaging studies have shown that autism spectrum disorders (ASDs) may be associated with abnormalities in brain structures and functions at rest as well as during cognitive tasks. However, it remains unclear if functional connectivity (FC) of all brain neural networks is also changed in these subjects. In this study, we acquired functional magnetic resonance imaging scans from 93 children with ASD and 79 matched healthy subjects. Group independent component analysis was executed for all of the participants to estimate FC. One-sample *t*-tests were then performed to obtain the networks for each group. Group differences in the different brain networks were tested using two-sample *t*-tests. Finally, relationships between abnormal FC and clinical variables were investigated with Pearson’s correlation analysis. The results from one-sample *t*-tests revealed nine networks with similar spatial patterns in these two groups. When compared with the controls, children with ASD showed increased connectivity in the right dorsolateral superior frontal gyrus and left middle frontal gyrus (MFG) within the occipital pole network. Children with ASD also showed decreased connectivity in the left gyrus rectus, left middle occipital gyrus, right angular gyrus, right MFG and right inferior frontal gyrus (IFG), orbital part within the lateral visual network (LVN), the left IFG, right precuneus, and right angular gyrus within the left frontoparietal (cognition) network. Furthermore, the mean FC values within the LVN showed significant positive correlations with total score of the Childhood Autism Rating Scale. Our findings indicate that abnormal FC extensively exists within some networks in children with ASD. This abnormal FC may constitute a biomarker of ASD. Our results are an important contribution to the study of neuropathophysiological mechanisms in children with ASD.

## Introduction

Autism spectrum disorders (ASDs) include a range of developmental disorders with a variety of clinical presentations characterized by impairments in the following: (1) social-communication and (2) restricted and repetitive behaviors and interests (American Psychiatric Association [APA], 2013). Furthermore, the diagnosis of ASD is grounded in presenting behavior and history of cognitive development (Bent et al., 2017). The prevalence rate of ASD has markedly increased over time, and ASD now affects nearly 1% of children in the worldwide (Gulati et al., 2017). The skyrocketing morbidity of ASD has resulted in enormous financial and emotional costs, as well as immense pressures on daily lives. Therefore, identification of the cause, accurate diagnosis, and early effective intervention are of vital importance to individuals with ASD and society as a whole (Li D. et al., 2017). Recently, more and more studies have begun to focus on changes in the brains of patients with ASD. However, the neurobiological bases underlying the symptoms and etiology in children with ASD are still unclear.

A growing number of neuroimaging studies have investigated functional or structural changes in the brains of people with autism. Magnetic resonance spectroscopy findings indicated that there was an excitatory/inhibitory imbalance in ASD patients (Hwang et al., 2017). PET and SPECT confirmed the disorder of neurotransmitters and glucose metabolism in patients with ASD (Zürcher et al., 2015; Mitelman et al., 2018). Furthermore, significant advances in MRI technology over the past few decades have greatly enriched our understanding of neuropathological differences in ASD. For example, Van Rooij et al. (2018) found that ASD was associated with decreased thickness in the temporal cortex and increased cortical thickness in the frontal cortex. Similarly, Kohli et al. (2018) found that children with ASD have an increased local cortical gyrification. Using functional magnetic resonance imaging (fMRI), Rausch et al. (2016) found that the entire amygdala had significantly reduced connectivity to visuospatial and superior parietal areas. Bednarz et al. (2017) found decreased functional connectivity (FC) between the left inferior frontal gyrus (IFG) and the left inferior occipital gyrus in ASD. In a task state fMRI study, Wadsworth et al. (2018) found increased activation in the left ventral premotor and right middle temporal gyrus during intransitive actions in ASD. Moreover, it is very interesting to note that local over-connectivity (in the posterior cingulate gyrus) and under-connectivity (in some visual regions) were observed in a study of local resting state FC in ASD (Nair et al., 2018). However, most previous studies were focused on the local level, and abnormal FC in all brain neural networks had not been well-understood and may be important neurobiological changes for ASD.

Independent component analysis (ICA) is a blind source separation technique that has been widely used to identify and quantify distribution area modes or spatial networks of related activities (Braden et al., 2017; Bernas et al., 2018; Bi et al., 2018). Additionally, it plays an important role in decomposing mixed data into independent components (ICs). Resting state networks can be found within ICs according to their spatiotemporal characteristics (Li C. et al., 2017). ICA is well suited to analyzing resting state functional magnetic resonance imaging (rs-fMRI) data without prior selection of seed regions. It has also been widely used in functional studies of healthy individuals (Smith et al., 2009) and patients with various diseases, such as primary insomnia (Li et al., 2018) and intractable epilepsy (Robinson et al., 2017). Using ICA, previous studies also found abnormal FC in patients with ASD (Cerliani et al., 2015; Itahashi et al., 2015; Nebel et al., 2016; Braden et al., 2017). For example, Braden et al. (2017) studied men aged 40–64 years with ASD and found decreased involvement of the cortico-striatum-thalamic-cortical neural network, and concluded that ASD men had less activity in brain networks that allow flexible thinking than typical men. Itahashi et al. (2015) studied the connection between different aspects of abnormal neuroanatomy in male ASD patients aged 19–50 years; their results suggested that changes in different aspects of brain morphology may occur simultaneously in specific brain networks in ASD. Cerliani et al. (2015) studied the intrinsic FC of brain networks in 166 male ASD patients aged 7–50 years and 193 age-matched normal male controls and found increased FC in primary sensory networks and subcortical networks. Furthermore, through extracting their interesting visual and motor regions using ICA, Nebel et al. (2016) found disrupted visual–motor FC in ASD children aged 8–12 years. However, most previous studies focused on FC in one or a few single regions or in one or a few interesting networks in ASD patients, but not in all brain neural networks simultaneously involved in oscillating activity. Moreover, many previous studies focused on adults or older children over 6 years old with ASD and little attention has been paid to younger children aged 2–5.5 years.

To better understand the neural mechanism and its relationship with clinical features of ASD, we used group ICA to research the FC of different neural networks in children with ASD. Three hypotheses were tested: (1) the ASD group and the healthy subjects group would have similar spatial patterns of the different neural networks; (2) the ASD group would demonstrate altered FC in some neural networks; and (3) the altered FC in some neural networks would have significant correlations with some clinical variables.

## Materials and Methods

### Subjects

Ninety-three outpatients aged 2–5.5 years from the Shenzhen Children’s Hospital, Shenzhen, China were recruited in this study from November 2016 to April 2018. All of the patients fulfilled DSM-V criteria for ASD, as diagnosed by clinical interview, and completed the Childhood Autism Rating Scale (CARS) and the Autism Behavior Checklist (ABC). The CARS and ABC are the main diagnosis and screening instruments for ASD in children currently available in china. The CARS was assessed by trained psychologists. It includes 15 items on the scale, and each item has four grades. A total score less than 30 indicates non-autism, a total score of 30–36 indicates mild to moderate autism, and a total score of 36 indicates severe autism. This scale is suitable for people over 2 years old. The ABC was completed by parents. It contains 57 items, and each item has four grades. A total score above 30 indicates the presence of suspected ASD symptoms, and a total score above or equal to 67 indicates the presence of ASD symptoms. This checklist is suitable for people aged 8 months to 28 years. Developmental Diagnostic Scale of Children Aged 0 to 4 years was adapted to assessment the develop quotient (DQ < 70 as low score). Seventy-nine matched healthy subjects, with no history of neurological or psychiatric disorder, were also included in the study. According to the recommendation of the Ethics Committee of Shenzhen Children’s Hospital, consent from the parents of the participants was obtained before the investigation. All of the children with ASD were scanned upon diagnosis and before treatment. Exclusion criteria included brain trauma, schizophrenia, hematological system diseases, any major medical illnesses, clinical diagnosis of neurologic diseases, history of psychiatric disorder, psychiatric family history, routine MRI examination showing abnormality, and any history of substance abuse in the subjects.

### Image Acquisition

Before scanning, all of the subjects were administered 0.5% chloral hydrate 0.5 ml/kg (maximum dose 10 ml) orally or via enema to induce and maintain sleep. Subjects continued sleeping during scanning, and were not stimulated.

MRI scanning was performed with a 3.0 T Siemens Skyra scanner in the Department of Radiology, Shenzhen Children’s Hospital. Subjects’ heads were fixed by foam pads to prevent head movement, and ears were blocked using cotton balls to prevent sleep disruption during scanning. Then, fMRI images were collected using T2-weighted gradient echo. Echo-planar imaging sequence parameters were as follows: repetition time (TR)/echo time (TE) = 2000 ms/30 ms, thickness/gap = 3.6 mm/0.72 mm, field of view = 230 mm × 230 mm, flip angle = 90°, matrix = 64 × 64, and slices = 35. Interleaved scanning alternated in the plus direction starting with odd-numbered slices. Thirty-five axonal slices including the whole brain were located approximately along the AC–PC line, and 240 volumes were obtained in approximately 8 min. After the MRI scanning, each participant’s images were inspected to ensure the images meet the experimental requirements. Meanwhile, a 3D magnetization-prepared rapid acquisition gradient echo (MPRAGE) T1-weighted sequence covering the whole brain (176 sagittal slices) was acquired. Corresponding acquisition parameters were set as TR = 2300 ms, TE = 2.26 ms, TI = 900 ms, flip angle = 8°, acquisition matrix = 256 × 256, FOV = 256 mm × 256 mm, and 1.00 mm slice thickness with a 0.5 mm inter-slice gap. In addition, we also obtained the T2-weighted images and T2-FLAIR images to preclude clinically silent lesions.

### Data Preprocessing

Data were preprocessed using the Graph Theoretical Network Analysis software package GRETNA_2.0^1^. Slice timing was first performed to correct for the differences in the acquisition time between different slices of a volume. The fMRI images for each subject were then realigned to correct for head movement using rigid-body transformation. Subjects with excessive head movement during the scan (>1.0 mm translation and/or 1.0° rotation) were to be excluded from further analysis. However, in the present study no subject was eliminated in this step. Next, the T1 image for each subject was co-registered with the functional images of the same subject. This co-registered T1 image was then segmented and normalized to the standard MNI space. The corresponding deformation parameters were then used to normalize the functional images to the MNI space. The resulting fMRI images were then spatially smoothed using a Gaussian Kernel of 6-mm full width at half-maximum (FWHM).

### Group ICA Analysis

All of the pre-processed images were then analyzed with the Group ICA Of fMRI Toolbox (GIFT^2^). We used spatial group independent component analysis (GICA) to obtain coherent patterns of activity (functionally connected brain networks). Data from all the subjects were first concatenated and ICs were extracted using the infomax algorithm. Finally, the fMRI images for all the subjects were decomposed into 20 spatially ICs. Next, in order to identify different brain network, we used the brain network templates provided in Smith et al. (2009). For a specific brain network, all the 20 resulting ICs were sorted by spatial correlation with the corresponding template, and the IC that had the highest correlation which best fit the template was selected, We were able to identified 9 brain networks for all the subjects: the medial visual network (MVN), occipital pole network (OPN), lateral visual network (LVN), default mode network (DMN), sensorimotor network (SMN), auditory network (AN), executive control network (ECN), right frontoparietal (perceived) network (FPN), and left frontoparietal (cognition) network (FPN). Furthermore, one-sample *t*-tests (*p* < 0.001, uncorrected) were performed for each network in the children with ASD and in the healthy subjects by using GIFT software. In addition, we conducted intergroup two-sample *t*-tests (*p* < 0.001) to compare the two groups, also using GIFT software. Then, multiple comparisons were performed by using the false discovery rate criterion to acquire the most significant results of intergroup two-sample *t*-tests (*p* < 0.05). Difference in gender between the two groups was assessed by a two-tailed Pearson’s chi-square test. A two-sample *t*-test was performed to compare the ages of the two groups. In addition, Pearson’s correlation analysis was performed to assess the relationships between the mean FC within each network showing significant differences and clinical variables (ABC, CARS, and DQ) in children with ASD.

## Results

### Demographic and Clinical Characteristics

The demographic characteristics of the subjects are shown in Table 1. There were no significant differences in gender (*P* = 0.053) or age (*P* = 0.089) between ASD and controls (Table 1). CARS, ABC, and DQ values met the ASD criteria. No corresponding scale measurement was conducted in healthy subjects.

**TABLE 1:** Demographic and clinical characteristics of Children with ASD and of healthy subjects

**Characteristic**	**ASD (*n* = 93)**	**Healthy subjects (*n* = 79)**	***p*-value**	***Chi-square /t value***
Gender (M/F)	83/10	62/17	0.053^*a*^	3.741
Age (years)	3.12 ± 1.16	3.49 ± 1.59	0.089^*b*^	–1.711
CARS	34.27 ± 2.02	–	–	–
ABC	67.66 ± 14.89	–	–	–
DQ	53.61 ± 8.15	–	–	–

*Values are mean ± SD. M, Male; F, Female. ^*a*^The *p*-value was obtained using a chi-square test. ^*b*^The *p*-value was obtained using a two-sample *t*-test.*

### Neural Networks in Children With ASD

**FIGURE 1:**
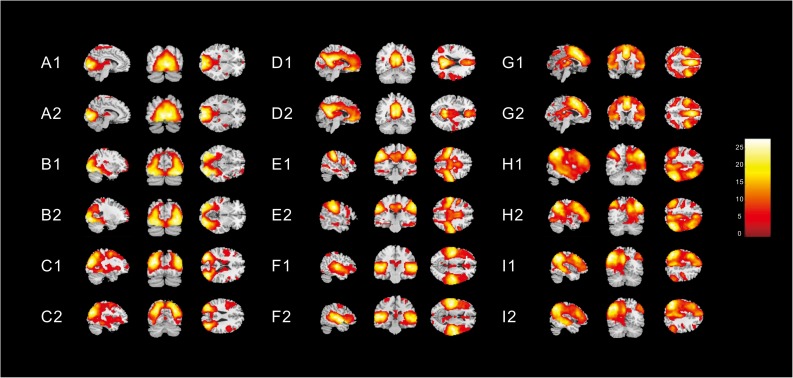
Spatial patterns of the nine different networks in (1) children with ASD and (2) in healthy subjects [one-sample *t*-tests (*p* < 0. 001)]. These images were based on a statistical parameter mapping of one-sample *t*-tests for each voxel non-zero contribution. (A) Medial visual network, (B) Occipital pole network (OPN), (C) Lateral visual network (LVN), (D) Default mode network, (E) Sensorimotor network, (F) Auditory network, (G) Executive control network, (H) right Frontoparietal (perceived) network (FPN), and (I) left FPN (cognition).

Nine different neural networks were identified in subjects: the MVN, OPN, LVN, DMN, SMN, AN, ECN, right FPN (perceived), and left FPN (cognition). The two groups had similar spatial patterns of the nine networks (one-sample *t*-tests, *p* < 0.001). Figure 1 shows the spatial patterns of the nine different networks in the children with ASD and in healthy subjects.

### Altered FCs of Nine Different Neural Networks in Children With ASD

**FIGURE 2:**
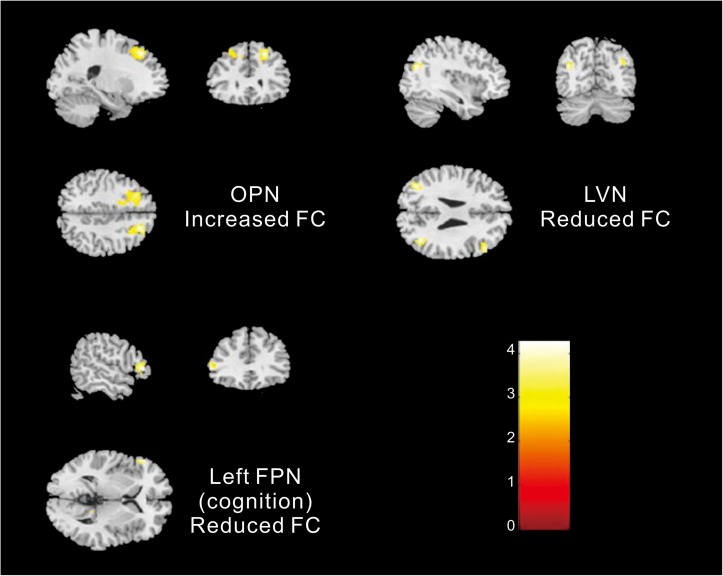
Altered FC within networks in children with ASD compared with healthy subjects [two-samples *t*-test (*p* < 0. 05, corrected)].

Different contributions from some brain regions in nine networks were detected in the children with ASD compared with healthy subjects. We found that children with ASD showed increased FC in the following regions: the right dorsolateral region of the superior frontal gyrus (SFGdor) and the left middle frontal gyrus (MFG) within the OPN. Meanwhile, decreased FC was found in the left gyrus rectus (REC), left middle occipital gyrus (MOG), right angular gyrus (ANG), right MFG and right IFG, orbital part (ORBinf) within the LVN, the left IFG, right precuneus (PCUN), and right ANG within the left FPN (cognition). These results are summarized in Figure 2 and Table 2.

**TABLE 2:** Significantly increased and reduced FC in neural networks of children with ASD compared with healthy subjects.

**Network**	**Increased or reduced (+/−)**	**Abnormal brain region**	**MNI (x, y, z)**	**Voxels**	***t* score**
OPN	+	SFGdor.R	(21, 30, 45)	128	5.34
	+	MFG.L	(−27, 24, 48)	187	5.04
LVN	−	REC.L	(0, 54, −15)	37	4.08
	−	MOG.L	(−39, 69, 27)	48	4.82
	−	ANG.R	(39, −63, 27)	62	4.79
	−	MFG.R	(36, 6, 39)	41	3.89
	−	ORBinf.R	(48, 27, 24)	86	4.76
Left FPN (cognition)	−	IFG.L	(−57, 33, 3)	32	4.85
	−	PCUN.R	(12, −45, 9)	29	4.76
	−	ANG.R	(54, −60, 30)	34	4.36

*OPN, occipital pole network; LVN, lateral visual network; left FPN (cognition), left Frontoparietal (perceived) network. SFGdor.R, right dorsolateral superior frontal gyrus; MFG.L, left middle frontal gyrus; REC.L, left gyrus rectus; MOG.L, left middle occipital gyrus; ANG.R, right angular gyrus; MFG.R, right middle frontal gyrus; ORBinf.R, right inferior frontal gyrus, orbital part; IFG.L, left inferior frontal gyrus; PCUN.R, right precuneus.*

### Relationships Between the FC Within the Different Networks and Clinical Variables in Children With ASD

**FIGURE 3:**
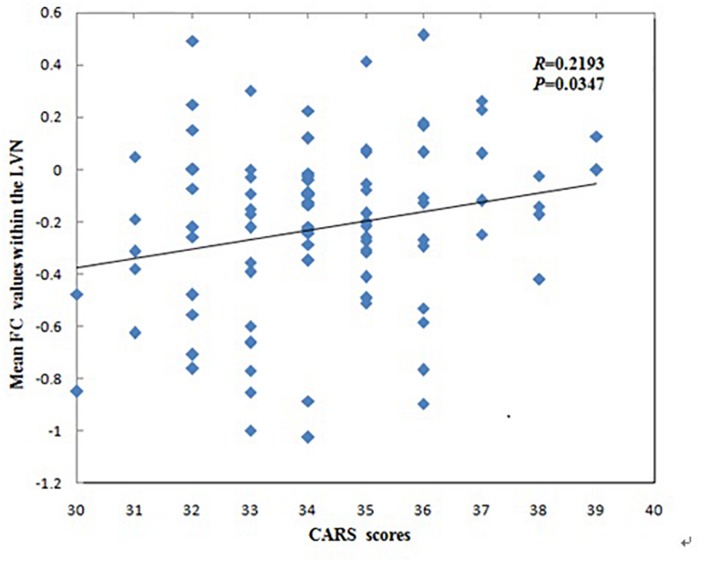
Correlations between mean functional connectivity (FC) values within the lateral visual network (LVN) and Childhood Autism Rating Scale scores in children with Autism Spectrum Disorder.

The mean FC values within the LVN showed positive correlations with CARS scores in children with ASD (*r* = 0.2193, *p* = 0.0347, Figure 3). No other significant correlations with any of the clinical variables were found for other networks showing significant connectivity differences.

## Discussion

In this study, using group ICA, we found nine different neural networks in ASD and healthy subjects, and the two different groups had similar spatial patterns of these networks. Furthermore, FC was altered within the OPN, LVN and left FPN (cognition) between the two groups. In addition, the mean FC values within the LVN showed positive correlations with CARS scores.

### Similar Spatial Patterns of Neural Networks

In this study, we found that the two different groups had similar spatial patterns of nine neural networks. Our finding supports the perspective that the functional architecture of the brain is very similar in different individuals and different mental states; this seems to be a stable characteristic of the brain (Smith et al., 2009; Geerligs et al., 2015). Furthermore, previous studies found that both during tasks and at rest, the functional network structures of the brain are intrinsic standard architecture (Cole et al., 2014; Bertolero et al., 2015; Schultz and Cole, 2016). As for studies of the brain’s functional architecture, Smith et al. (2009) suggested that neural connections are the basis of functional networks and exist regardless of their functional activity at any given time. In this study, our findings suggest that there are similar spatial patterns of neural networks in children with ASD and in healthy subjects; these patterns may be the basis of functional networks.

### Behavioral Consequences of Abnormal FC Within the Neural Networks in ASD

Altered FCs within the OPN and LVN were detected in the current study. The OPN and LVN belong to visual areas, and are associated with sensory processing (Farrant and Uddin, 2016; Nebel et al., 2016). A previous study reported that up to 96% of individuals with ASD exhibit atypical sensory characteristics (Hand et al., 2017). Some psychological theories about autism suggest that atypical sensory processes are central features of autism (Paakki et al., 2010). The large-scale dysfunction of FC in the brain of autistic people has been described most conclusively in terms of the excessive or inadequate sensitivity to perceptual stimuli (Rausch et al., 2016). We found that the abnormal FC brain regions in the OPN include the MFG and SFGdor, which are associated with social and communication disorders in ASD patients (Assaf et al., 2010). The MFG is primarily in charge of the coordination of different information (Japee et al., 2015). Previous studies have also shown that information processing of ASD patients is incomplete, and they are unable to integrate and process information, and unable to communicate normally in public places (Skoyles, 2011; Mohd et al., 2015). Meanwhile, the abnormal FC brain regions in LVN include the REC, MOG, ANG, MFG, and ORBinf. The REC and ORBinf belong to the IFG, which plays an important role in social interaction (Bi et al., 2018). The ANG act as an across-modal center for combining and integrating multisensory information, redirecting attention to key information, understanding environmental events, manipulating mental representations and solving problems (Pua et al., 2018). The MOG belongs to the occipital lobe, whose main executive function is associated with visual perception construction and motion perception (Johnson et al., 2015; Bi et al., 2018). Since social functioning requires one to select and integrate many social perceptual stimuli simultaneously, to some extent the abnormal perceptual processing may explain some social difficulties in ASD patients (Rausch et al., 2016). These abnormal FC regions are associated with social dysfunction and communication difficulties in ASD patients. Thus, our results provide an explanation for social dysfunction and communication disorders in ASD patients from the perspective of neurological function.

Altered FC within the left FPN (cognition) was also detected in the current study. We found decreased connectivity in the left IFG, right PCUN, and right ANG within the left FPN (cognition). The maps of the left FPN (cognition) are consistent with Broca’s and Wernicke’s areas, which are known for language lateralization (Smith et al., 2009), and have generally been implicated in semantic processing and lexical access, respectively (Maximo et al., 2017), and play a crucial role in social cognition (Igelström et al., 2017). Our findings were consistent with those of previous studies, where children and adolescents with ASD showed decreased FC in Broca’s area, while adults with ASD showed decreased FC in Wernicke’s area (Lee et al., 2017). A task-state fMRI study reported that children with ASD showed hypo-connectivity within the left hemisphere language network irony comprehension (Williams et al., 2013). Furthermore, as a core feature of ASD, language and communication deficits often extend to loss in reading abilities, especially dyslexia (Bednarz et al., 2017; Maximo et al., 2017). Our study found abnormal FC in left FPN (cognition), which may be related to deficiencies in language, semantic processing, contextual understanding, long-term social functioning, and communication in children with ASD.

### The Relationships Between Mean FC and Clinical Variables in ASD Patients

The mean FC values within the LVN that correspond to cognition–space (Smith et al., 2009) showed positive correlations with CARS scores in ASD patients. This may indicate a greater FC value within the LVN and more serious clinical symptoms in ASD patients. The LVN is mainly located in the lateral occipital cortex, which not only plays a crucial role in integrating visual information and visual and tactile object recognition, but also in tasks involving motor performance and tactile stimuli, especially involving body movements (Johnson et al., 2015). Our finding is similar to that of a previous study, where alterations in high-level visual processing regions (one of the “autism-specific structural networks”) in ASD was significantly correlated with the social interactions and stereotypical behavior scores of the Autism Diagnostic Observation Schedule (Grecucci et al., 2016). Thus, this correlation might suggest that FC values within the LVN may be related to clinical symptoms such as the inability to initiate or respond to social interaction, or gazing at light or movement. This correlation may be a standing neurobiological explanation for the clinical symptoms that mentioned above of the disorder.

We found that children with ASD showed decreased FC in the left REC, left MOG, right ANG, right MFG and right ORBinf within the LVN, however, the mean FC values within the LVN showed positive correlations with CARS scores in children with ASD. The possible explanation for this contrary trend may be their different principles of these two analyses. The principle of ICA algorithm is to use the independence of the source signal to estimate the ICs through the linear transformation of a group observation signals (Mi, 2014; Xie et al., 2017). It is assumed that multiple brain regions are unrelated and the signal components decomposed by ICA are independent of each other. In this study, it shows the alteration of FC values in various unrelated brain regions within the LVN. However, correlation coefficient is a statistical indicator of the degree of close correlation between two random variables. Pearson’s correlation analysis is a commonly used linear correlation analysis. In our study, it shows the corresponding relationship between the mean FC values within the LVN and CARS. Another possible explanation for this may be the different severity of clinical symptoms in different ASD patients. Uneven severity of the participants might influence the results in this study. A previous study found that the alterations in high-level visual processing regions were significantly correlated with the social interactions and stereotypical behavior scores of the Autism Diagnostic Observation Schedule in ASD (Grecucci et al., 2016). The severity of clinical symptoms may be an important role in the study of ASD, so further studies are needed to explore the effects on FC by dividing the severity of clinical symptoms of ASD.

Autism spectrum disorder include a range of developmental disorders with average onset at 2–4 years of age (American Psychiatric Association [APA], 2013). In this study, we used group ICA to research the FC in young children aged 2–5.5 years with ASD. Using other study methods, previous studies also found abnormal FC in young children with ASD (Shen et al., 2016; Wang et al., 2017; Chen et al., 2018). For example, a recent study exploring FC in young boys aged 3.47–7.9 years with ASD revealed that under-connectivity within social and cognitive association areas was related to social deficits and over-connectivity within sensorimotor and visual regions associated with restricted and repetitive behaviors (Chen et al., 2018). Wang et al. (2017) studied the FC in boys aged 3.47–7.93 years with ASD and found that left post-central gyrus was significantly more positive with right ANG. Shen et al. (2016) studied the FC in children with the mean age of 3.5 years with ASD and found that, compared with typically developing controls, the ASD group had significantly weaker connectivity between amygdale and some brain regions. Obviously, the results of previous studies on peers with ASD are also greatly inconsistent with each other. Therefore, there may be other influence factors besides age. Previous studies (American Psychiatric Association [APA], 2013; Wang et al., 2017; Chen et al., 2018) have shown that ASD include a range of atypical neurological disorders which may be caused by aberrant developmental trajectories, and suggested that different ASD patients show varieties of atypical spatial distribution in distinct neural circuits. And even the same neural circuit show different atypical spatial distribution in different patients with ASD (Hahamy et al., 2015; Chen et al., 2018). These different patterns might lead to different behavioral characteristics and contribute to atypical FC within ASD. However, the lack of a comprehensive understanding of impairment in children with ASD is one of the limitations of current research.

The present study has several limitations. First, our study was cross-sectional and all participants were preschool-aged children. Therefore, the interpretation of our results cannot be extended to other stages of development. Longitudinal datasets with dense sampling are needed for a more complete description of the precise nature of developmental trajectories of FC networks, and would be sensitive to subtle changes over time within functional brain networks in ASD throughout life. Second, future studies should also include additional indicators of ASD severity that are currently unavailable in ASD groups to better understand the relationship between clinical manifestations and the altered FC of some brain regions. Finally, we only investigated connectivity alterations within the nine different neural networks in ASD patients. However, it might be useful to examine alterations in inter-networks in ASD patients to better understand the neural substrates of underlying symptoms.

As the first attempt to explore FC alterations within the nine different neural networks associated with ASD and healthy controls, this study demonstrates the possible disruption of the OPN, LVN and left FPN (cognition) networks in children with ASD. Dysfunction of these networks may constitute biomarkers for the diagnosis of ASD, and clarifying this functional change may further our understanding of the neural substrates of symptoms in ASD patients from the perspective of internal network connectivity.

## Data Availability

All datasets generated for this study are included in the manuscript and/or the supplementary files.

## Ethics Statement

This study was carried out in accordance with the recommendations of the Ethics Committee of Shenzhen Children’s Hospital with written informed consent from parents of all subjects. All parents of subjects gave written informed consent in accordance with the Declaration of Helsinki. The protocol was approved by the Ethics Committee of Shenzhen Children’s Hospital.

## Author Contributions

GJ, SX, JL, BL, ML, and LW conceived and designed the experiments. SX, CY, YG, PL, and XM acquired the data. XF, MY, BY, and WH performed the clinical data. SX, JL, LW, BL, and YW analyzed the clinical data. SX, ML, and GJ wrote the manuscript. All authors reviewed the manuscript and approved it for submission.

## Conflict of Interest Statement

The authors declare that the research was conducted in the absence of any commercial or financial relationships that could be construed as a potential conflict of interest.

## Footnotes

1 http://www.nitrc.org/projects/gretna/

2 http://mialab.mrn.org/software/gift/
